# Tocilizumab for the Treatment of Mevalonate Kinase Deficiency

**DOI:** 10.1155/2018/3514645

**Published:** 2018-08-26

**Authors:** Nadia K. Rafiq, Helen Lachmann, Frodi Joensen, Troels Herlin, Paul A. Brogan

**Affiliations:** ^1^Infection and Inflammation and Rheumatology Section, University College London Great Ormond Street Institute of Child Health, 30 Guilford Street, London WC1 E1H, UK; ^2^National Amyloidosis Centre, University College London Division of Medicine, London, UK; ^3^National Hospital of the Faroe Islands, J. C. Svabos Gøta, Tórshavn 100, Faroe Islands; ^4^Department of Paediatrics, Pediatric Rheumatology Clinic, Palle Juul-Jensens Boulevard 99, 8200 Aarhus N, Denmark

## Abstract

Mevalonate kinase deficiency (MKD) is a severe autoinflammatory disease caused by recessive mutations in MVK resulting in reduced function of the enzyme mevalonate kinase, involved in the cholesterol/isoprenoid pathway. MKD presents with periodic episodes of severe systemic inflammation, poor quality of life, and life-threatening sequelae if inadequately treated. We report the case of a 12-year-old girl with MKD and severe autoinflammation that was resistant to IL-1 and TNF-*α* blockade. In view of this, she commenced intravenous tocilizumab (8 mg/kg every 2 weeks), a humanised monoclonal antibody targeting the IL-6 receptor (IL-6R) that binds to membrane and soluble IL-6R, inhibiting IL-6-mediated signaling. She reported immediate cessation of fever and marked improvement in her energy levels following the first infusion; after the fifth dose, she was in complete clinical and serological remission, now sustained for 24 months. This is one of the first reported cases of a child with MKD treated successfully with tocilizumab and adds to the very limited experience of this treatment for MKD. IL-6 blockade could therefore be an important addition to the armamentarium for the treatment of this rare monogenic autoinflammatory disease.

## 1. Introduction

Mevalonate kinase deficiency (MKD) is a rare autosomal recessive autoinflammatory disease caused by mutations in MVK, causing loss of function of the enzyme mevalonate kinase (MVK). Broadly, two clinical phenotypes are recognized that vary in severity as determined by the level of residual enzyme activity. The milder phenotype of MKD is a periodic fever syndrome characterized by frequent episodes of fever typically lasting 3–7 days, abdominal pain, lymphadenopathy, inflammatory eye disease, rashes, and arthralgia [1] and typically associated with intracellular MVK activity greater than 1% [2]. The more severe phenotype is mevalonic aciduria (MA), usually associated with MVK enzyme activity less than 0.5%. MA may result in a high rate of stillbirth; survivors present soon after birth with severe systemic inflammation, facial dysmorphism, severe failure to thrive, developmental delay, seizures, and hepatic involvement [3]. Worldwide, approximately only 300 patients with MKD have been reported [4], including a recent large series from Europe [1].

The pathophysiology of autoinflammation in MKD is poorly understood and is suggested to occur as a result of loss of synthesis of isoprenoid lipids downstream of MVK [5], in particular geranylgeranyl diphosphate. This latter is necessary for prenylation (the addition of a hydrophobic compound) of small GTPases, the loss of which causes activation of the inflammasome and release of IL-1*β* [6]. More recent studies have indicated a central role for the pyrin inflammasome as the driver of autoinflammation in MKD [7]. There is an inverse relationship between RhoA activation and activation of the pyrin inflammasome: RhoA activation induces downstream kinases, pyrin phosphorylation, and inhibitory binding of phosphorylated pyrin by 14-3-3 proteins [7]. In MKD, deficient geranylgeranylation of RhoA renders it inactive. This is because RhoA activation is dependent on its translocation to the plasma membrane, and this membrane targeting of RhoA is dependent on geranylgeranylation at its C-terminus [7]. Since geranylgeranyl pyrophosphate, the substrate of geranylgeranylation, is a product of the mevalonate pathway, loss-of-function mutations of MVK results in depletion of geranylgeranyl pyrophosphate, failure of Rho membrane binding, and thus persistent RhoA inactivation and consequently pyrin activation which induces IL-1*β* overproduction in myeloid cells [7, 8]. Thus mutations of MVK lead to release of the normal, constitutive tonic inhibition of the pyrin inflammasome and excess IL-1 production [7].

Consequently, attempts to treat MKD have mainly focused on IL-1 blockade, as reflected in recent international expert consensus guidance [9]. In fact, therapeutic success with IL-1 blockade is at best modest. For anakinra (recombinant interleukin-1 receptor antagonist, blocking IL-1*β* and IL-1*α*), a retrospective series [10] reported only 30% complete remission, and 70% partial remission in patients with MKD treated with daily anakinra. Canakinumab, a monoclonal antibody against IL-1*β*, appears to have superior efficacy to anakinra in retrospective studies, with up to half of patients achieving complete response [10–12]. A major prospective randomised controlled trial of canakinumab (150 mg monthly) for MKD has reported that 35% met a definition of response at 16 weeks; and 45% had a physician global assessment of <2/10 [13]. Since it is now increasingly apparent that MKD is in fact a multicytokine-driven disease, with involvement of other proinflammatory cytokines including IL-6 and tumour necrosis factor*-α* (TNF-*α*) [14, 15], this somewhat limited efficacy of IL-1 blockade is perhaps unsurprising. It is thus clear that whilst IL-1 blockade may be useful in some patients with MKD, alternative treatments for those who fail to respond adequately to IL-1 blockade are urgently required.

Tocilizumab is a humanised, monoclonal, antihuman IL-6 receptor (IL-6R) antibody that binds to membrane and soluble IL-6R, inhibiting IL-6-mediated signaling [16]. Tocilizumab binds to soluble IL-6R present in serum and joint fluid as well as membrane-bound IL-6R expressed on the surface of cells, leading to inhibition of receptor-mediated IL-6 signaling and suppression of several physiological roles of IL-6 [16, 17]. Experience of using tocilizumab for the treatment of MKD is limited, with (to date) only six cases reported. We therefore add to these limited data describing a child with MKD who was successfully treated with tocilizumab having failed other treatments and review the literature regarding IL-6 blockade in MKD.

## 2. Case Presentation

We present the case of a twelve-year-old girl originally from the Faroe Islands with compound heterozygotic MKD (p.V377I/c.417insC), who first presented with symptomatic periodic fever attacks from the age of 3 months. These presented as recurrent episodes of fever (39–41°C) without infectious cause, occurring once or twice monthly, associated with rigors, pallor, fatigue, lymphadenopathy (inguinal, axillary, and intra-abdominal), abdominal pain, oral ulceration, and arthralgia/myalgia of the lower limbs. Attacks lasted between 3 and 7 days and were accompanied by very high acute phase responses (C reactive protein [CRP] typically greater than 100 mg/L). Attacks were also triggered by vaccinations. Her past medical history included an episode of Stevens–Johnson syndrome in response to penicillin at the age of three years and appendectomy aged 7 years of a normal appendix. She was referred to us in London at the age of 12 years. At that time, she was receiving the anti-TNF*α* agent etanercept, which she had been on for the previous 34 months. She had at best only partial response to etanercept in terms of attack severity and duration, but was still missing 100 days per year of school because of attacks which occurred twice a month, lasting for 3 days. In addition, despite etanercept, her inflammatory markers remained significantly raised between attacks: CRP 82 mg/L (reference range [RR] < 10); serum amyloid A (SAA) 1310 mg/L (RR < 10), indicative of severe systemic inflammation in-between attacks and significant risk of reactive AA amyloidosis. Anakinra (2 mg/kg/day; recombinant interleukin-1 receptor antagonist) had also been tried previously, but was complicated both by a severe skin rash and also by the worst disease flare she had ever experienced; hence after four weeks, this was discontinued. Following a six-week washout period from the etanercept (during which she suffered one severe attack), in December 2015 she started intravenous tocilizumab 8 mg/kg every 2 weeks.

**Figure 1:**
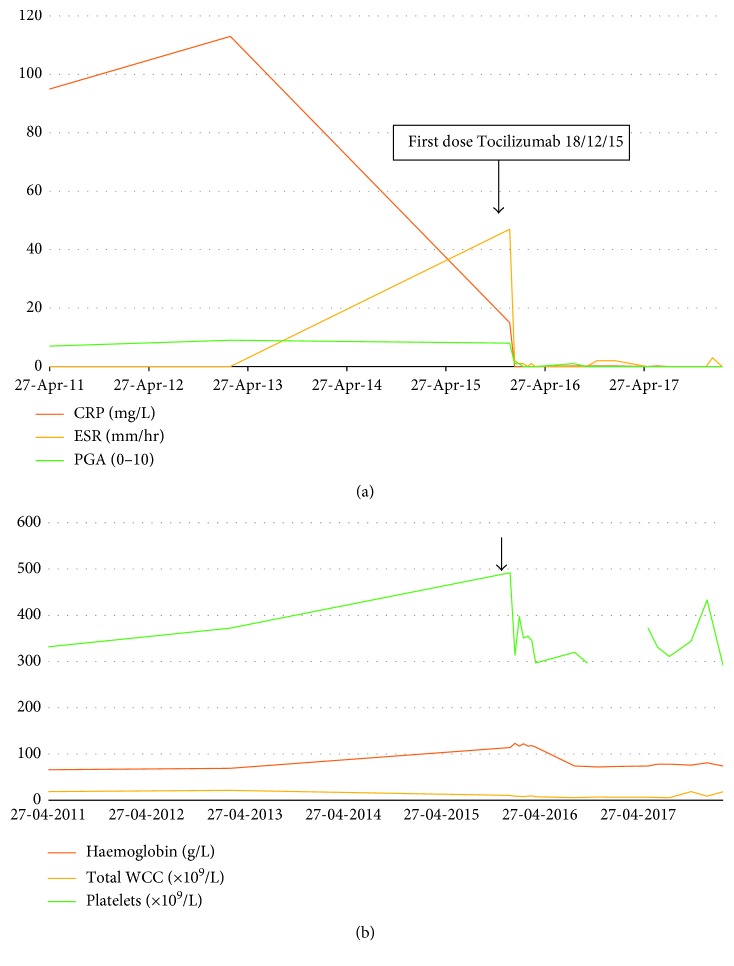
(a) Change in clinical disease activity inflammatory markers in response tocilizumab. *Note*. PGA: physician global assessment, 0–10, 0 indicating absence of disease activity and 10 indicating severe disease. ESR: erythrocyte sedimentation rate. CRP: C reactive protein. PGA fell from 8/10 immediately before the first tocilizumab infusion to 1/10 when assessed immediately prior to the next infusion 2 weeks later. This excellent clinical and serological response has been sustained for 24 months. (b) Change in haemoglobin, total white cell count, and platelet count in response to tocilizumab. Black arrow shows date of first dose of tocilizumab.

After one dose of tocilizumab, her CRP and ESR both rapidly normalised (Figure 1(a)). She had one minor attack with minor, short-lived fleeting rash, but no fever after the first dose. Since normalisation of CRP is a well-known effect of tocilizumab and may occur without necessarily any true improvement in clinical disease activity, we also prospectively used a physician global assessment of disease activity using a 0 to 10 scale, 0 indicating no disease activity and 10 indicating severe disease activity (Figure 1(a)), and change in haemoglobin, white cell count, and platelets (Figure 1(b)) as adjunctive laboratory indicators of successful treatment. In addition, the patient reported significant improvement even after a single dose of tocilizumab, with improved energy levels, reduction in pain, and improvement in oral ulceration. She was also able to return to full-time school.

This excellent clinical and serological response has been sustained for more than 24 months. There have been no reported adverse events. The patient was switched to weekly subcutaneous tocilizumab at 162 mg in June 2016 for ease of administration and reduction in hospital attendances. A year since that change, the patient has had several short-lived flares lasting 1–3 days associated with fever, adenitis, and mouth blistering, but she has not required prednisolone as before and overall expressed a preference for the subcutaneous route as it was associated with improved quality of life despite the breakthrough fever attacks.

## 3. Discussion

There is no definitive treatment for MKD, and the current rationale for treatment is based on anecdotal case reports, retrospective series, and a limited (albeit increasing) knowledge regarding the pathogenesis of autoinflammation in MKD. The European project, Single Hub and Access point for pediatric Rheumatology in Europe (SHARE), was launched to address this important unmet clinical need and provided a systematic literature review and expert consensus for the treatment of MKD and other autoinflammatory diseases [9]. It is currently suggested that patients with MKD may benefit from treatment with NSAIDs, steroids, and biologics, particularly IL-1 blockade or etanercept. Furthermore, the SHARE guidance emphasizes that statins and colchicine are not effective in MKD [9]. Upon failure of IL-1 blockade and etanercept, IL-6 blockade should be the next biologic instigated, before considering haematopoietic stem cell transplantation (HSCT) [9], although data supporting the use of tocilizumab in MKD are very limited. As such, our case represents (to the best of our knowledge) only the seventh subject with MKD treated with tocilizumab. The efficacy we observed was immediate, dramatic, and sustained over 24 months. This suggests that IL-6 blockade should be given more consideration in patients with MKD, especially after failure of IL-1 blockade is demonstrated.

The biggest series of patients with MKD was recently described from the Eurofever registry [1] providing additional, albeit retrospective data regarding the treatment of MKD. Of note, in that series, 27/114 patients received anakinra: 6/28 (22%) were reported as complete responders; 18/27 (67%) were partial responders; and 11% had no response to anakinra. Anakinra doses are not described in detail in this report, but are likely to be an important factor influencing this (at best) modest efficacy [18]. Canakinumab was given to 5/114 patients and resulted in complete remission in 4/5, with a partial response in the remaining subject [10]. Etanercept was used in 27/114 patients and had a beneficial effect in 16/27 patients (59%), of whom only two (7%) had a complete response [10]. Eleven/27 patients (41%) failed to respond to etanercept [10]. Of note, only 2 patients with severe MKD received tocilizumab, but the treatment responses are not described in any detail in this series [10]. Whilst expert consensus places tocilizumab as an important third-line biologic treatment for MKD [9], there have only been six MKD cases treated with this agent reported worldwide [15,19–21]. The details of these reports are summarized in Table 1.

The pathogenesis of autoinflammation in MKD is poorly understood although recent studies have emphasized that, unlike cryopyrin-associated periodic fever syndromes (CAPS; a purely IL-1-driven autoinflammatory disease), MKD is probably a multicytokine-driven monogenic autoinflammatory disease [2, 5, 15]. One mechanistic model of inflammation in MKD is that of enhanced IL-1*β* release as a result of decreased Rho/Rac GTPase prenylation, particularly in hyperthermic conditions [5]. Stoffels et al., however, observed an increased cytokine response from cells from MKD patients specific for TLR4, TLR2, and NOD2 stimulation, which included not only IL-1*β*, but also IL-1*α*, TNF*α*, and IL-6 [15]. Based on this observation, they questioned whether or not IL-1*β* is the central driving cytokine in the pathogenesis of MKD. Since they also observed excessive caspase-1 protein in leucocytes from MKD patients, they postulated that this could provide an explanation as to why these cells are more easily triggered to secrete IL-1*β*, but emphasized also that other proinflammatory cytokines are involved. They concluded that this could account for the fact that not all patients respond to blockade of IL-1, as emphasized by the aforementioned results of the only randomised controlled trial published to date [13]. These observations, and the emerging (albeit limited) clinical experience of IL-6 blockade in MKD (Table 1), argue strongly for consideration of IL-6 blockade in MKD, particularly for those who fail to respond adequately to IL-1 blockade [15], but perhaps also for a clinical trial exploring dosing and efficacy of tocilizumab as first-line choice of biologic for MKD. From a practical perspective, tocilizumab is significantly cheaper than canakinumab and avoids the need for daily injections as is the case of anakinra.

The dose and route of administration of tocilizumab for MKD has varied across the published reports (Table 1). Shendi et al. [19] used tocilizumab at an intravenous dose of 8 mg/kg, every four weeks. This is the NICE approved dose for the treatment of rheumatoid arthritis [22] and for some forms of JIA in children weighing ≥30 kg [23]. This patient went into remission with normalisation of inflammatory markers, but had recurrent upper respiratory tract infections (well described with tocilizumab) [24] necessitating a 50% dose reduction. This lower dose was inadequate to control the MKD however, and was therefore increased again to 7 mg/kg four weekly, with reasonable disease control [19]. We did not observe any infections in our patient, who was treated with 8 mg/kg tocilizumab every two weeks (as per a typical systemic JIA dosing regimen [25]), or latterly whilst on weekly subcutaneous tocilizumab. Regarding this latter point, there are no data that suggest any benefit over intravenous or subcutaneous administration; clearly subcutaneous administration has potential practical advantages, such as avoiding the need for hospital admission for intravenous administration as was the case for a patient.

In conclusion, we report a dramatic and sustained (24 months) clinical and serological response to tocilizumab in a 12-year-old child with MKD, which to the best of our knowledge represents only the seventh patient reported. Since a recent randomised placebo-controlled clinical trial of IL-1 blockade has suggested that more than 50% of patients may fail to respond adequately, we suggest that further consideration should be given to alternative approaches such as IL-6 blockade, particularly for those individuals who fail IL-1 blockade, and especially before considering haematopoietic stem cell transplantation as a therapeutic option.

## Figures and Tables

**Table 1:** Previous reports of tocilizumab for the treatment of MKD.

	Shendi et al. (*n* = 1) [19]	Stoffels et al. (*n* = 2) [15]		Lane et al. (*n* = 2) [20]		Muster et al. (*n* = 1) [21]
Patient	1	2	3	4	5	6

TOC dose (mg/kg) and route of administration	7 IV	8 IV	8 IV	8 IV	8 IV	8 IV

Frequency of administration (weeks)	4	4	4	4	4	4

Age (range), years at onset of TOC	13	Not described	Not described	24	13	36

Treatment prior to TOC	COL	ANA	ANA	ANA	ETA	NSAID
	PRED			ETA		SIMVA
	ETA					ANA
	ANA					

Duration of treatment (months)	20	5	5	24	13	48–60

Outcome						
Clinical	CR	CR	CR	CR		PR
Serological	CR	Not described	PR	CR		—

Adverse events	URTI	Not described	Not described	Not described		Not described

Comments	CR at dose of 8 mg/kg but due to adverse events dose reduced, ultimately with stable clinical and serological status on 7 mg/kg IV every 4 weeks	—	—	MKD complicated by AA amyloidosis. Remained on therapy with PRED 0.5 mg/kg/day	Stabilized on monotherapy with TOC	After starting TOC, average hospital admissions dropped 11/yr to 3/yr
						TOC given in combination with IVMP for first 3 yrs then as monotherapy

IV: intravenous; COL: colchicine; PRED: prednisolone; ETA: etanercept; ANA: anakinra; NSAID: nonsteroidal anti-inflammatory drug; SIMVA: simvastatin; CR: complete response; PR: partial response; URTI: upper respiratory tract infection; TOC: tocilizumab; yrs: years.
